# Peripheral polyneuropathy and acute psychosis from chronic nitrous oxide poisoning: A case report with literature review

**DOI:** 10.1097/MD.0000000000028611

**Published:** 2022-08-05

**Authors:** Radhika Sood, Thibault Parent

**Affiliations:** Department of Internal Medicine, Hôpitaux Universitaires de Genève, HUG, Geneva, Switzerland.

**Keywords:** acute psychosis, case report, nitrous oxide, peripheral polyneuropathy

## Abstract

**Rationale:**

Nitrous oxide (NO) is a commonly used drug in medical practice, restoration, and the automobile industry. Recreational abuse is an emerging public health problem owing to its accessibility and drug properties.

**Patient concerns:**

A 25-year-old male was hospitalized with acute psychosis and lower-extremity sensorimotor proprioceptive ataxia due to nitrous oxide abuse.

**Diagnosis:**

Laboratory studies confirmed a vitamin B12 deficiency. Magnetic resonance imaging of the spinal cord showed normal findings. Electrophysiological testing confirmed length-dependent sensorimotor polyneuropathy, with a predominant motor component and axonal degeneration.

**Intervention and outcomes:**

Abstinence from toxic substances was suggested, and vitamin B12 substitution was introduced. The patient was lost to follow up.

**Lessons:**

Nitrous oxide toxicity is multisystemic and is thought to result from vitamin B12 inactivation. Recent case reports postulated direct paranodal lesions resulting from nitrous oxide consumption. Neurological, neuropsychiatric, and hematological toxicities are among those explored in this case report. Correction of the functional vitamin B12 status and nitrous oxide abstinence are essential in the treatment process.

**Take home messages**

- Nitrous oxide is a commonly used drug, and recreational abuse is an emerging public health problem due to its accessibility and drug properties.
- The toxicity of nitrous oxide is multisystemic and is thought to result from vitamin B12 inactivation, possibly resulting in paranodal lesions.
- This case report explores toxic sensorimotor polyneuropathy occurring without myelopathy as well as acute psychosis in a young adult following recreational abuse of nitrous oxide.
- Differential diagnoses included posterior spinal cord syndrome, subacute inflammatory demyelinating polyneuropathy, and subacute combined degeneration of the spinal cord due to copper and vitamin E deficiencies.
- Treatment through correction of the functional vitamin B12 status is essential, and studies have postulated treatment with cobalamin and possibly methionine supplementation.

## 1. Introduction

### 1.1. Nitrous oxide: anesthetic and analgesic effects

Nitrous oxide, also known as dinitrogen oxide or dinitrogen monoxide, is a commonly used drug in the medical practice, in restauration (antibacterial effect allowing for use in the preparation of whipped cream), as a fuel booster in the motor industry (automobile racing and rocket engines^[1]^) and recreationally.

Its use as an anesthetic is thought to result from noncompetitive inhibition of the NMDA subtype of glutamate receptors, which is also responsible for its dissociative effects.^[2]^ This analgesic effect is thought to be due to the inhibition of supraspinal GABA-A receptors and concomitant activation of spinal GABA-A receptors.^[3]^ Commonly used in medical practice, it is, for example, one of the first-line treatments for vaso-occlusive crisis in patients with sickle cell anemia.^[4]^

According to a narrative review published by the European Society of Anesthesiology in 2019, nitrous oxide is considered the least soluble and fastest acting inhaled anesthetic agent available.^[5]^ While the risk profile has been subject to debate in the past, the ENIGMA-II trial, a large randomized controlled trial published in 2015, did not show increased cardiovascular risk following nitrous oxide anesthetic use.^[6]^

### 1.2. Recreational use of N_2_O

While medical use is predominant, nitrous oxide abuse constitutes an emerging global public health problem, as discussed by Bao et al in a recent publication.^[7]^ Commonly known by the street name of “whippets,” “laughing gas,” “hippy crack,” or “sweet air,” it is one of 5 most frequently used inhalants,^[8]^ and described as the second most popular drug, following cannabis in the UK, according to a study published in 2015.^[9]^ Recreational abuse is often integrated into the context of polydrug abuse.^[10]^

Recreational users commonly purchase 8-g containers of highly pressurized nitrous oxide, known as “whippets”. Cost varies with current estimates in the United Kingdom of approximately £2.7 when ordered online. The gas was transferred to balloons to allow consumption, providing approximately 8 L at standard room temperature and pressure. Inhalation directly from ‘whippets” comprises the risk of intraalveolar rupture leading to pneumomediastinum and subcutaneous emphysema due to the high pressure.^[1,11]^

The increase in recreational use of nitrous oxide can be attributed to its low cost, legal status, rapid euphoric, reversible effect, achieved within 10 s.^[11,12]^

This case report highlights the clinical manifestations of extensive nitrous oxide abuse, a pathology rarely encountered in Switzerland.^[13]^

## 2. Case presentation

This case report explores a 25-year-old male who was hospitalized from 19.05.2020 to 09.06.2020 in the Internal Medicine Department of Geneva University Hospital.

### 2.1. Primary concerns and symptoms of the patient

The patient was referred to the emergency room following lipothymia. He complained of dysesthesia, hypoesthesia, and heightened paraesthesia in a stocking-and-glove distribution with recent proximal irradiation of his lower limbs. Recently, he complained of difficulty walking.

His medical history revealed no recent infections. The patient returned from a holiday in the United States on the same day, where he had resided for the past 3 months. He described many festive nights with regular sleep deprivation (4 hours on average as compared to his normal 8 hours) and an increase in the consumption of toxic substances. He reported regular cigarette and cannabis smoking (10–15 joints daily over the past 6 months, regular consumption for the past 5 years), and alcohol abuse (10 units daily for the past 3 months). Nitrous oxide consumption patterns were described as 200–300 balloons daily for the past 2–3 months, with the last inhalation on May 17.05.2020, and an alleged consumption of 100 balloons thrice weekly prior to this. He had attempted cocaine for the first time 3 weeks prior, which he had not repeated since.

### 2.2. Medical history

The patient’s medical history was significant for 2 inaugural epileptic fits in the past month, described as generalized, following increased alcohol consumption. Further workup was proposed upon consultation but was not followed up on. Psychiatric history included 2 major depressive episodes in 2016 and 2018, with intermittent manic phases according to collateral history.

### 2.3. Clinical findings

His vital signs, cranial nerve examination, and sphincter function were unremarkable. A long-tract neurological examination revealed pathological findings. Sensory examination revealed tactile hypoesthesia of the lower limbs in a stocking distribution ascending to the knees, which also affected her fingers. Vibration sensation was evaluated as normal in the metacarpophalangeal joints (7/8) and decreased bilaterally in the hallux (5/8). The positional sense was pathological on the left hallux but normal on the right side. Heel-to-shin testing was pathological and worsened with the eyes closed. Motor evaluation revealed bilateral distal paresis with a strength of 4/5 bilaterally. Deep tendon patellar reflexes were described as 3+ and were not obtained bilaterally at the Achilles tendon. Gait was compromised, described as ataxic with irregular step length, with posture revealing an unstable stance and an increased sustentation polygon. The patient did not present with any radicular pain.

Psychiatric evaluation revealed a hypervigilant, anxious patient who was oriented to all spheres (person, place, and time). His speech accelerated and he presented with intact comprehension and repetition. Episodic and remote memories were intact. The patient presented with persecutory delusions, as well as visual, auditory, and kinesthetic hallucinations, of which he was partially critical. He described being watched by people with harmful intentions, with covered faces and weapons (Kalashnikovs), who were cackling him from the corridor and telling him that they would hurt his spinal cord. He further claimed that a recent knee injury that occurred while he attempted to escape from a sniper was responsible for his difficulty in walking. He did not report any depressive symptoms or recent periods of elation.

To summarize, the examination revealed marked sensorimotor proprioceptive ataxia of the lower extremities with acute psychosis.

### 2.4. Investigations

Laboratory analysis showed macrocytic normochromic anemia with vitamin B12 deficiency. One carbon metabolism marker, homocysteine, was not detected. Complete blood count revealed an increase in hypersegmented neutrophils. Peripheral blood swab analysis showed slight anisocytosis of red blood cells but no morphological stigmata of B12 deficiency.

Serological evaluation excluded acute viral hepatitis B and C, HIV infection, and borreliosis. A lumbar puncture was performed, and the results were normal. Antiganglioside antibodies were not detected in the serum or CSF.

MRI of the nervous system did not reveal any signal anomalies, specifically no sign supporting myelopathy or cortico-subcortical lesions, when considering movement artifacts.

Somatosensory evoked potentials, obtained by electrical stimulation of the tibial nerve bilaterally, revealed prolonged latency at the popliteal point and in the lumbar region, suggestive of peripheral lesions. No cortical or subcortical response was observed.

Electroneurography revealed diminished amplitudes in the left sural nerve and bilaterally in the peroneal nerve. Sensitivity testing of the right sural and left radial nerves was normal. Myography revealed a collapsed M response amplitude for the peroneal nerve (when tested at the left quadriceps femoris muscle) and bilaterally for the tibial nerve (when tested at the abductor hallucis muscle). Thus, electrophysiological testing confirmed length-dependent sensorimotor polyneuropathy with a predominant motor component and axonal and myelin degeneration.

### 2.5. Differential diagnosis

The patient was diagnosed with toxic sensorimotor polyneuropathy caused by nitrous oxide, alcohol abuse, and severe vitamin B12 deficiency. A large differential diagnosis was established. Posterior spinal cord syndrome due to vitamin B12 deficiency and compressive myelopathy due to loss of proprioception and vibratory sensation was discussed and excluded following spinal MRI. Subacute inflammatory demyelinating polyneuropathy was ruled out because of the lack of proximal muscle involvement and subsequent electrophysiological examination. Other causes of subacute combined degeneration of the spinal cord, including copper and vitamin E deficiencies, were excluded. Causes other than nitrous oxide and alcohol abuse for vitamin B12 deficiency include malnutrition, and there is no argument for pernicious anemia.

### 2.6. Treatment

The therapeutic measures proposed to the patient included definitive cessation of toxins (nitrous oxide and alcohol), vitamin B12 substitution, and neurological rehabilitation. Vitamin B12 was prescribed daily for 5 days, followed by heparin administration for 4 weeks and monthly administration for 6 months.

### 2.7. Outcome and follow-up

The patient was discharged and lost to follow up.

## 3. Discussion and conclusion

### 3.1. Nitrous oxide toxicity: vitamin B12

Nitrous oxide causes irreversible oxidation of cobalt, which renders vitamin B12 bivalent and inactive.^[14]^ This inhibits myelin synthesis, leading to demyelination, axonal swelling, and ultimately, loss.^[15]^

Vitamin B12 is primarily obtained from meat, fish, and dairy products. However, the human body is incapable of endogenous production. Absorption occurs in the ileum following binding of intrinsic factors in the duodenum. The active form of vitamin B12 contains cobalt in the reduced monovalent form. Active cyanocobalamin is involved in 2 essential in vivo reactions: conversion of L-methylmalonyl coenzyme A into succinyl coenzyme A and transmethylation of homocysteine which results in methionine. Transmethylation is essential for the synthesis of DNA and the myelin sheath.

Serum cobalamin levels correlate poorly with tissue levels and do not exclude cobalamin deficiency. One-carbon metabolic markers, including methylmalonic acid and homocysteine, were used as surrogate markers. Methylmalonic acid is considered more sensitive than homocysteine to B12 deficiency, as vitamin B9 deficiency can also cause elevated homocysteine levels.^[16]^ The latter allows for functional assessment of B12 status.^[7,17]^

Some case reports have postulated that nitrous oxide toxicity may be independent of vitamin B12 deficiency.^[18]^ Tani et al postulated that toxicity in the paranodal region is independent of the inhibition of methionine metabolism, that is vitamin B12 deficiency.^[19]^

### 3.2. Clinical toxicity of nitrous oxide abuse

A literature review found multiple case series exploring the toxicity of nitrous oxide abuse; the population concerned was predominantly young.^[1,16,20]^ Garakani et al performed a systematic review of 91 individual cases, of which 72 reported neurologic sequelae and 11 reported psychiatric effects, published in 2016. Low to normal vitamin B12 levels were found in 52 of 91 cases.^[1]^

Neurological sequelae were further explored by Oussalah et al in a 2019 meta-analysis of 100 patients.^[16]^ This study found that subacute combined degeneration was described in 28% of patients with nitrous oxide abuse, which comprises combined peripheral neuropathy and myelopathy. Reduced methionine synthesis results in demyelination, prominent in the dorsal column of the spinal cord, affecting the corticospinal tract and posterior funiculus, which leads to gliosis.^[21]^ Impairment of the posterior columns causes loss of position and vibration sense, while damage to the corticospinal tract results in weakness and spasticity. The first case report describing subacute combined degeneration associated with intermittent nitrous oxide use dates back to 1978, and multiple reports have been published since then.^[7,14,20–32]^

Myelopathy alone due to dorsal column pathology was the second most common neurological complication in 26% of patients. The latter manifests as loss of proprioception in a stocking distribution.^[16]^ This has been described as a Guillain-Barré-like syndrome.^[33,34]^ Other rare neurological presentations described in the literature include recurrent paraparesis,^[35]^ atypical subacute combined degeneration associated with involuntary movements such as dystonia and pseudoathetosis,^[36]^ and optic atrophy.^[37]^ More severe neurologic complications have been reported with nitrous oxide exposure in patients with rare congenital genetic deficiencies.^[3]^ Retrospective reviews exploring the electrodiagnostic features found in nerve conduction studies have shown mixed axonal and demyelinating neuropathies.^[7,38]^

Neuropsychiatric manifestations have long been associated with vitamin B12, including memory loss, depression, hypomania, dementia, and paranoid psychosis with auditory and visual hallucinations. The latter comprises “megaloblastic madness.”^[8,39,40]^ Nonetheless, Nagele et al suggested the potential of nitrous oxide in treatment-resistant depression in a blinded, placebo-controlled crossover trial that included 20 patients.^[41]^

Other relevant clinical effects include hematological toxicity, pancytopenia, and hyperhomocysteinemia.^[42]^ The latter is a known thrombotic risk factor owing to increased coagulability.^[21]^ Dermatological manifestations have often been described as hyperpigmented macular patches on the trunk.^[11,43]^ Furthermore, anecdotal deaths have been associated with nitrous oxide consumption.^[44]^

### 3.3. Treatment approach

Treatment through correction of the functional vitamin B12 status is essential, as it represents a potential reversible cause of myelopathy.^[23]^ Studies have postulated treatment with Cbl and possibly methionine supplementation.^[36,45]^ Various case series have shown notable neurological improvements with excellent prognoses.^[37]^

## 4. Conclusions

This case report explores toxic sensorimotor polyneuropathy occurring without myelopathy as well as acute psychosis in a young adult following recreational abuse of nitrous oxide. This allows for further understanding of the neurological and neuropsychiatric complications of nitrous oxide toxicity. The limitations of this study include the loss of the patient to follow-up, prognostic factors, and treatment response, which have not been explored in detail.

### Author contributions

R.S. drafted the manuscript. Both authors revised the manuscript and approved the final manuscript.

### Acknowledgments

We thank the internal medical team responsible for managing the patient.
